# An open label, single-armed, exploratory study of apatinib (a novel VEGFR-2 tyrosine kinase inhibitor) in patients with relapsed or refractory non-Hodgkin lymphoma

**DOI:** 10.18632/oncotarget.23806

**Published:** 2018-01-02

**Authors:** Ling Li, Sa Xiao, Lei Zhang, Xin Li, Xiaorui Fu, Xinhua Wang, Jingjing Wu, Zhenchang Sun, Xudong Zhang, Yu Chang, Feifei Nan, Jiaqin Yan, Zhaoming Li, Mengyuan Shi, Ken H. Young, Mingzhi Zhang

**Affiliations:** ^1^ Department of Oncology, Lymphoma Diagnosis and Treatment Center of Henan Province, The First Affiliated Hospital of Zhengzhou University, Zhengzhou, China; ^2^ Department of Oncology, The First Affiliated Hospital of Zhengzhou University, Zhengzhou, China; ^3^ Department of Hematopathology, The University of Texas MD Anderson Cancer Center, Houston, TX, USA

**Keywords:** apatinib, non-Hodgkin lymphoma, angiogenesis, VEGFR-2, clinical trial

## Abstract

**Background:**

Apatinib, a novel small molecule vascular endothelial growth factor receptor-2 (VEGFR-2) tyrosine kinase inhibitor, have shown remarkable efficacy in many solid cancers. But evidence of antitumor activity in patients with lymphoma is still limited. We conducted an open-label, single-armed, exploratory study in relapse or refractory non-Hodgkin lymphoma patients for the efficacy and safety of apatinib.

**Experimental design:**

Patients with relapse or refractory non-Hodgkin patients meet the criteria were eligible for enrollment. Treatment comprised of oral apatinib 500 mg once daily with 21 days as a treatment cycle. The primary end point was response rate. Secondary end points included progression-free survival (PFS) and overall survival (OS).

**Results:**

Between February 2016 and December 2016, 21 patients were enrolled. The ORR (CR plus PR) was 47.6% (10 of 21 patients) included 9.5% CRs and 38.1% PRs. 23.8% patients achieved stable disease made the DCR 71.4% (15/21). The median OS was 7.3 months (95% CI, 7.1 to 7.9) and the median PFS was 7.1 months (95% CI, 4.2 to 7.3). Most patients suffered from grade 1 to grade 2 treatment-related adverse events and the most common nonhematologic adverse events were proteinuria (47.6%), hypertension (42.9%) and hand-foot syndrome (33.3%), respectively.

**Conclusions:**

In our study, the results we presented showed apatinib might have a rapid, safe and high efficacy on relapsed or refractory non-Hodgkin lymphoma patients. Based on the data more clinic trials are expected to be taken to identification the efficacy of apatinib on lymphoma further.

## INTRODUCTION

Lymphoma is the 12th most common cancer incidence in China, and it ranks the ninth in the mortality rate among males and fifteenth among females, respectively [1]. Non-Hodgkin lymphoma encompasses many kinds of cancers, including 85–90% lymphomas arise from B lymphocytes with the rest derive from T or NK lymphocytes. Mainstays of treatment for lymphoma have been chemotherapy or radiotherapy. Meanwhile monoclonal antibody therapies such as Rituximab, Alemtuzumab and Ofatumumab play a very important role on treat of this disease [2]. However, there is no standard treat therapy for patients with relapse or refractory non-Hodgkin lymphoma. Though many patients are in need of rescue therapies, few available agent and chemotherapy tolerance are barriers for the treatment of these patients.

As a novel small molecule vascular endothelial growth factor receptor-2 (VEGFR-2) tyrosine kinase inhibitor, apatinib could strongly inhibit new angiogenesis in tumor tissue by highly selective compete the ATP binding site to block downstream signal transduction [3]. Anti-angiogenesis effect has been proven to be effective in many solid tumors according to several clinical trials, especially in advanced gastric carcinoma [4–8]. However, evidence of antitumor activity leading to improved response rate or survival in patients with relapse or refractory non-Hodgkin lymphoma is still limited. Herein, we reported 21 cases of advanced lymphoma and evaluated the safety and efficacy of apatinib in relapse or refractory non-Hodgkin lymphoma patients.

## MATERIALS AND METHODS

### Patients

Relapse or refractory patients age between 18 and 70 years had to have taken at least two lines of chemotherapy fail before participating in the study with histologically confirmed non-Hodgkin lymphoma were eligible for enrollment. Treatment failure was defined as intolerable adverse effects or disease progression during treatment with chemotherapy. All the pathological results were confirmed by at least two experienced clinical pathologists. Additional enrollment criteria were as follows: at least one measurable lesion, Eastern Cooperative Oncology Group (ECOG) performance status of 0 to 2, and acceptable hematologic, hepatic, and renal function. Patients with uncontrolled blood pressure with medication (> 140/90 mmHg), those with bleeding tendency, and those receiving thrombolytics or anticoagulants, were excluded. Patients were also excluded if they had central nervous system involvement. All patients provided written informed consent before participating in the study.

### Study design and treatment

This study was an open label, single-armed, exploratory study undertaken in China. Patients who met the eligibility criteria would take oral apatinib 500 mg once daily with 21 days as a treatment cycle until they experienced disease progression or intolerable toxicity or withdrew consent from the study. Treatment interruption resulting from toxicities was allowed for no more than 7 days (either continuously or cumulatively) in each treatment cycle. Efficacy was evaluated every two cycles and once every three months after six cycles. The primary end point was response rate. Secondary end points included progression-free survival (PFS) and overall survival (OS).

### Study assessments

Baseline evaluation included history and physical examination, CT scans, routine laboratory studies, bone marrow evaluation, cardiac ultrasonography and ECG. Lesions were evaluated by CT and colour Doppler ultrasonography every two cycles and once every three months after six cycles until disease progression or dead. Adverse events (classified and graded using Common Terminology Criteria for Adverse Events [version 3.0]) were assessed at baseline (after patients provided written informed consent) and 1st day of each cycle until at least 21 days after off the drug.

### Statistical analysis

The primary end point was response rate. The secondary end points were overall (OS) and progression-free survival (PFS). PFS was calculated from the date of enrollment to the date of progression or death from any cause. OS was calculated from the date of enrollment to the date of death or the last follow-up. OS and PFS were estimated with standard errors and their 95% confidence intervals (CI) were estimated using the Kaplan–Meier method to account for censoring. Statistical analyses were performed with SPSS version 21 (SPSS, Inc., Chicago, IL, USA).

## RESULTS

### Patient characteristics

Between February 2016 and December 2016, 21 patients were enrolled. The median age at the start of oral treatment was 56 years (age ranges from 20 to 67 years) with the following distribution: DLBCL in 11 cases, FL in 1 case, MCL in 2 cases, PTCL in 3 cases, ENKT in 4 cases.

The male/female ratio was 1.63:1. Of the cases, 11 (52.4%) had diagnosed stage III–IV disease. Systemic B symptoms were present in 8 patients (38.1%) and 10 patients (47.6%) had elevated LDH levels. Baseline characteristics of patients were listed in Table 1.

**Table 1:** Baseline patient characteristics

Characteristics	Number of patients
	(*n* = 21)
Age, years	
Median	56
Range	20 to 67
Gender	
Male	13
Female	8
Histology	
DLBCL	11
FL	1
MCL	2
PTCL	3
ENKT	4
Stage	
I–II	10
III–IV	11
B-symptoms	
No	13
Yes	8
Serum LDH	
Normal	11
Elevated	10
Bone marrow involvement	
No	18
Yes	3
serum β2 microglobulin	
Normal	12
Elevated	9

Abbreviations: DLBCL, diffuse large B cell lymphoma; FL, follicular lymphoma; MCL, mantle cell lymphoma; PTCL, peripheral T-cell lymphoma; ENKT, Extranodal NK/T-cell lymphoma, nasal type; LDH, lactate dehydrogenase.

### Efficacy

**Figure 1:**
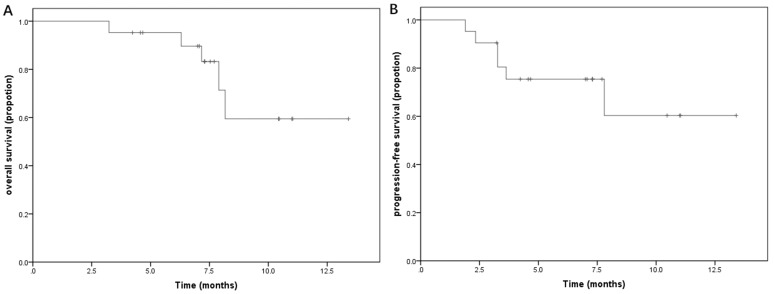
Kaplan-Meier estimates of (**A**) progression-free (PFS) and (**B**) overall survival (OS). (A) Median PFS 3.5 months (95% CI, 2 to 5). (B) Median OS 6 months (95% CI, 5.0 to 8.5).

Efficacy assessment of all 21 patients showed the ORR (CR plus PR) was 47.6% (10 of 21 patients) included 9.5% CRs and 38.1% PRs. 23.8% patients achieved stable disease made the DCR 71.4% (15/21) (Table 2). The median OS was 7.3 months (95% CI, 7.1 to 7.9) and the median PFS was 7.1 months (95% CI, 4.2 to 7.3) (Figure 1).

**Table 2:** Response rates

Response	Number of patients (%)
	*n* = 21
CR	2(9.5)
PR	8(38.1)
SD	5(23.8)
PD	6(28.6)
ORR	10(47.6)
DCR	15(71.4)

Abbreviation: CR, complete response; PR, partial response; SD, stable disease; PD, progressive disease; ORR, overall response Rate; DCR, disease control rate.

### Safety

**Figure 2:**
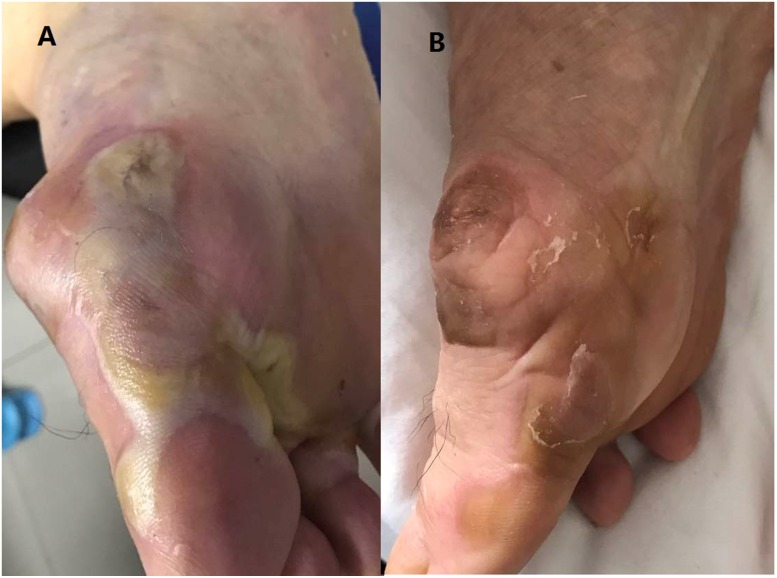
The grade 4 hand-foot syndrome (**A**) Taking apatinib for 6 weeks. (**B**) A week off the durg.

Adverse events (AEs) were assessed in all patients, including haematological and non-haematological toxicities (Table 3). In our study, most patients suffered from grade 1 to grade 2 treatment-related AEs and the most common non-hematologic AEs were proteinuria (47.6%), hypertension (42.9%) and hand-foot syndrome (33.3%), respectively. One grade 4 hand-foot syndrome was observed and the symptom was relieved obviously after the drug was stopped for 3days (Figure 2). Grade 3/4 leucopenia occurred in 2 patients and grade 3/4 anaemia in 1 patient. Fatigue was a common manifestation among leucopenic patients.

**Table 3:** Safety of apatinib in relapse/refractory non-Hodgkin lymphoma

Adverse Event	NO. (%)	
	any grade	≥ grade 3
Hematologic		
Leukopenia	10(47.6)	2(9.5)
Neutropenia	5(23.8)	0(0)
Anemia	4(19.0)	1(4.8)
Thrombocytopenia	5(23.8)	0(0)
Non-hematologic		
Hypertension	9(42.9)	1(4.8)
Hand-foot syndrome	7(33.3)	1(4.8)
Proteinuria	10(47.6)	2(9.5)
Headache	1(4.8)	0(0)
Hyperbilirubinemia	4(19.0)	1(4.8)
Fatigue	5(23.8)	0(0)
Diarrhea	2(9.5)	0(0)

## DISCUSSION

The necessity of angiogenesis has been proved in many physiologic and pathologic processes of cancer, peripheral arterial or chronic inflammatory disease. Angiogenesis plays a very important role in tumor proliferation and metastatic dissemination [9]. Tumor tissue needs to rely on the new blood vessels to provide oxygen and nutrients to meet the needs of proliferation of the tumor cells. Signal transduction pathway induced by VEGF with VEGFR plays a vital role in accommodating cell proliferation and tumor angiogenesis. Tumor angiogenesis, mediated by VEGF, has been validated to be a potential therapeutic target in cancer treatment. The addition of inhibitors of VEGF or its receptor to standard treatment has lengthened PFS in many solid tumors. Bevacizumab and sunitinib have improved overall survival in advanced colorectal and renal cancers, respectively [10, 11].

VEGF is known to be a mediator of angiogenesis in non-Hodgkin lymphoma, increased levels predict a poor response to treatment [12, 13]. The VEGFR family includes VEGFR-1(Flt-1), VEGFR-2(KDR/Flk-1), VEGFR-3(Flt-4), NRP-1 and NRP-2 with VEGFR-2 is supposed to be closer to generation of tumor vessels [14]. Bevacizumab could significantly lengthen median PFS from 10.4 to 20.7months (*P* = .007) compared with single-agent rituximab group in relapsed or refractory follicular lymphoma [15]. Even though the addition of bevacizumab increased toxicity compared with single-agent rituximab and caused serious adverse events in several patients, the regimen appeared to be better tolerated than the standard chemotherapy/rituximab combinations used to treat follicular lymphoma. In the SWOG 0515 trial, the addition of bevacizumab to standard chemotherapy/rituximab combinations showed no difference in statistic among the newly diagnosed DLBCL. [16]. Therefore, bevacizumab is not recommended to combine with R-CHOP. However, the lack of efficacy in the first-line therapy of DLBCL cannot be generalized to the treatment of other lymphomas. The immunomodulator lenalidomide also can inhibit angiogenesis, and showed remarkable efficacy with acceptable AEs in relapsed or refractory indolent non-Hodgkin lymphoma in a phase II trial. More studies are expected [17], oral lenalidomide monotherapy produced durable responses with manageable adverse events in patients with relapsed or refractory indolent NHL, warranting further investigation of treatment for indolent NHL.

The process of angiogenesis is mainly affected by microenvironment, and VEGF is the most important angiogenesis regulation factor. As an oral small-molecule tyrosine kinase inhibitor, apatinib could highly inhibit VEGFR-2, which is the main VEGFR regulating angiogenesis. The migration and proliferation of endotheliocytes related to VEGF is prevented and the formation of tumor neonatal microvessels is reduced, thus resulting in strong anti-tumor effect. This encouraging efficacy has been reported in several solid tumors [18, 19].

There are several preclinical and clinical studies have demonstrated the efficacy and safety of apatinib in solid tumors. A phase III trial evaluated the efficacy and safety of apatinib in advanced gastric carcinoma, showed a vast benefit in median OS (6.5 vs 4.7 months; HR, 0.709; 95% CI, 0.537 to 0.937; *P* = 0.0156) and median PFS (2.6 vs 1.8 months; HR, 0.444; 95% CI, 0.331 to 0.595; *P* < 0.001) compared with the placebo group. The adverse events were acceptable although dose modifications occurred in 21% patients on account of toxicity. [20]. Another multicenter phase II study of apatinib in metastatic triple-negative breast cancer (mTNBC) compared efficacy and safety of two different doses (750 mg/d vs 500 mg/d) and revealed that low dose is recommended other than high dose. [6], These data demonstrate that apatinib has a significant effect and can be well tolerated as a salvage therapy. Given few trial focused on patients with lymphoma, we managed a study to reveal the efficacy and safety of apatinib in lymphoma patients. Considering that dose modifications were very common in a Phase III trial which apatinib (850 mg/d) was recommended, a lower dose (500 mg/d) was applied to the cases in our study. And toxicities were generally well tolerated for the dose of 500 mg/d. Although there was 1 grade 3 hematologic toxicity case, more likely associate with chemotherapy.

In summary, as the tyrosine kinase inhibitor of VEGFR-2, apatinib have shown remarkable efficacy in many solid cancers [21]. The result of our study presented that apatinib might have a rapid, safe and high efficacy on lymphoma patients, DLBCL in particularly. The results of this study are provocative. however, the power of the conclusions is limited by the small study size. More prospective clinic trials are expected to be taken to select patients who can benefit from apatinib and identify the efficacy on lymphoma further.
